# Psychological Recovery 5 Years After the 2004 Niigata-Chuetsu Earthquake in Yamakoshi, Japan

**DOI:** 10.2188/jea.JE20130097

**Published:** 2014-03-05

**Authors:** Kazutoshi Nakamura, Kaori Kitamura, Toshiyuki Someya

**Affiliations:** 1 Division of Preventive Medicine, Niigata University Graduate School of Medical and Dental Sciences, Niigata, Japan; 1 新潟大学大学院医歯学総合研究科環境予防医学分野; 2 Department of Psychiatry, Niigata University Graduate School of Medical and Dental Sciences, Niigata, Japan; 2 新潟大学大学院医歯学総合研究科精神医学分野; 3 Niigata Institute for Traumatic Stress, Mental Health and Welfare Association in Niigata Prefecture, Niigata, Japan; 3 新潟こころのケアセンター（新潟県精神保健福祉協会）

**Keywords:** epidemiologic studies, earthquakes, mental health, psychological distress, social support

## Abstract

**Background:**

The 2004 Niigata-Chuetsu earthquake of Japan caused considerable damage. We assessed long-term changes in psychological distress among earthquake victims during the period 5 years after the earthquake.

**Methods:**

The participants were people aged 18 years or older living in Yamakoshi, a community in Niigata Prefecture near the epicenter. A self-administered questionnaire survey was conducted annually for 5 consecutive years after the earthquake. Response rates were 1316/1841 (71.5%) in 2005, 667/1381 (48.3%) in 2006, 753/1451 (51.9%) in 2007, 541/1243 (43.5%) in 2008, and 814/1158 (70.3%) in 2009. The questionnaire asked about demographic characteristics, including sex, age, employment status, social network, and psychological status. Psychological distress was assessed using the 12-item General Health Questionnaire and was defined as a total score of 4 or higher.

**Results:**

The overall prevalence of psychological distress decreased (*P* < 0.0001) gradually from 2005 (51.0%) to 2008 (30.1%) but tended to increase from 2008 to 2009 (*P* = 0.1590). Subgroup analyses showed that prevalence did not decrease over the 5-year study period among participants with poor social contact (*P* = 0.0659). From 2008 to 2009 prevalence increased in women (+7.5%, *P* = 0.0403) and participants aged 65 years or older (+7.2%, *P* = 0.0400).

**Conclusions:**

The prevalence of psychological distress in Yamakoshi people decreased steadily during the 4 years immediately after the earthquake but appeared to increase thereafter. The earthquake victims are still reestablishing their lives. Thus, continued attention should be focused on maintaining and further assessing their mental health.

## Abstract (ja)

**背景：:**

2004年に発生した中越地震は多くの被害をもたらした。本研究の目的は地震後５年間の被災者における心理的苦痛の長期的変化を評価することである。

**方法：:**

対象者は１８歳以上の山古志（震源近くの自治体）の住民であった。地震後の５年間に自記式質問票による調査が毎年行われた。回答率に関して、2005年は1316/1841（71.5%）、2006年は667/1381（48.3%）、2007は753/1451（51.9%）、2008年は541/1243（43.5%）、2009年は814/1158（70.3%）であった。質問票により、性、年齢、職業、社会的ネットワーク、精神状態に関する情報を得た。心理的苦痛は General Health Questionnaire１２項目版 （GHQ-12）を用いて評価し、合計点４点以上を心理的苦痛ありと定義した。

**結果：:**

全体として、心理的苦痛の有病率は2005年（51.0%）から2008年（30.1%）に徐々に低下した （P<0.0001）が、2008年から2009年は上昇傾向 （P=0.1590）であった。サブグループ解析により、社会的コンタクトの悪いグループでは5年間の有病率は低下が見られなかった （P=0.0659）。また、女性（+7.5%, P=0.0403）および65歳以上の群（+7.2%, P=0.0400）では、2008年から2009年の有病率の上昇が有意であった。

**結論：:**

山古志住民の心理的苦痛は地震後４年間に徐々に低下したが、その後は上昇傾向であった。地震被災者の生活の復興は本研究期間を超えて続いており、彼らのメンタルヘルスは引き続き評価・維持されるべきである。

## INTRODUCTION

The 2004 Niigata-Chuetsu earthquake in Japan had a near-epicenter maximum seismic intensity of 7 on the Japan Meteorological Agency’s Intensity (JMAI) scale and killed more than 60 people. Approximately 103 000 people sought refuge, and 16 000 houses were destroyed.^1^ A unique characteristic of the Niigata-Chuetsu earthquake was that mountainous areas experienced considerable damage on the ground and disruption of both traffic and communication networks.^2^ Such a large earthquake adversely affects the psychological status of people. After the Niigata-Chuetsu earthquake, various short-term psychiatric problems and symptoms of psychological distress were documented among the victims.^3^

Psychological problems appear to persist long after earthquakes. Previous epidemiologic studies found that the prevalence of psychological distress remains elevated many years after earthquakes.^4^^,^^5^ Similar long-standing adverse effects on psychological health may have been present after the Niigata-Chuetsu earthquake. Thousands of people in the disaster area required temporary housing for 3 years,^6^ and financial problems due to property damage and/or unemployment after the earthquake were long-lasting.^2^ Thus, the long-term effects of psychological distress after the Niigata-Chuetsu earthquake warrant investigation.

The village of Yamakoshi (currently part of Nagaoka City) was located near the epicenter of the earthquake, where physical damage was enormous. All village residents sought refuge in a nearby city after the event. To care for these people, annual health checks were conducted for 5 consecutive years after the earthquake. In this setting, the psychological status of Yamakoshi residents was also evaluated. The aim of this study was to assess long-term changes in psychological distress among the resident population during the 5 years after the Niigata-Chuetsu earthquake.

## METHODS

### Participants

The Niigata-Chuetsu earthquake occurred on October 23, 2004 and measured M 6.8 on the Richter scale. The epicenter was located at latitude 37°29′ N and longitude 138°87′ E.^7^ This survey was conducted by the local government and the Niigata Institute for Traumatic Stress (Mental Health and Welfare Association in Niigata Prefecture) as part of post-earthquake health checks for people registered as Yamakoshi residents between 2005 and 2009. All residents aged 18 years or older were targeted. Response rates to the survey were 1402/1841 (76.2%) in 2005, 729/1381 (52.8%) in 2006, 841/1451 (58.7%) in 2007, 594/1243 (47.9%) in 2008, and 883/1158 (77.3%) in 2009. After excluding people with missing data on age, sex, or psychological status, the final response rates were 1316/1841 (71.5%) in 2005, 667/1381 (48.3%) in 2006, 753/1451 (51.9%) in 2007, 541/1243 (43.5%) in 2008, and 814/1158 (70.3%) in 2009. Although the response rate differed by year, 224 participants participated in all 5 surveys. This subgroup was also analyzed for comparison with the results of whole-population analyses.

### Procedure

Self-administered questionnaires for the years 2005 through 2008 were delivered and collected by hand (for participants living in temporary refugee housing) or sent and collected by mail (for other participants). The 2009 questionnaires were collected by hand from all participants. The questionnaires elicited demographic information on sex, age, employment status, social network, and psychological status. Regarding social networks, participants were asked, “Do you easily talk with or call on neighbors in the community?”. Answers were categorized as “often,” “sometimes,” “not often,” or “rarely.” Participant psychological status was assessed using the 12-item General Health Questionnaire (GHQ-12). This study received approval from the Ethics Committee of the Niigata University School of Medicine.

### GHQ-12

The outcome of this study was level of psychological distress, as measured by the previously validated Japanese version of the GHQ-12.^8^^,^^9^ The GHQ is a self-administered tool originally developed as a screening tool for non-psychotic psychiatric illnesses^10^ and is used in clinical settings and in epidemiologic studies of mental health status in general populations. Participants are asked to assess their moods, feelings, and behaviors during the previous 4 weeks, and the tool is used to evaluate their responses on a 4-point response scale. All GHQ-12 items were rated using the GHQ method (0-0-1-1) before being summed to a total score (range, 0–12). A person with a high GHQ score is considered to have a high level of psychological distress, including depression, anxiety, and other psychiatric symptoms. Honda et al reported that a score of 4 or higher had satisfactory sensitivity and specificity as a GHQ-12 cutoff score in the Japanese general population; we therefore used this value.^9^

### Statistical analysis

The study outcome was prevalence of psychological distress, as defined by a GHQ-12 score of 4 or higher. The secular trend in the prevalence of psychological distress during the 5-year study period was observed and tested using the Cochran-Armitage test for trend. Secular trends were investigated by sex and age group, eg, elderly people (≥65 years of age) versus younger people (≤64 years of age). Secular trends were also investigated according to job status (unemployed vs other) and level of social contact (often/sometimes vs not often/rarely) for the respective years, because lack of employment and loss of contact in the community were reported to be strongly associated with psychological distress in a cross-sectional study of the Niigata-Chuetsu earthquake.^11^ The χ^2^ test was used to compare prevalence rates between 2 groups. Multiple logistic regression analysis was used to determine if sex (1, female; 0, male), age (1, ≥65 years; 0, ≤64 years), employment status (1, unemployed; 0, other), and social contact (1, not often/rarely; 0, often/sometimes) were independently related to the outcome variable. Data were analyzed utilizing SAS statistical software (release 9.1.3, SAS Institute Inc., Cary, NC, USA). A *P* value of less than 0.05 was considered to indicate statistical significance.

## RESULTS

Table 1 shows the characteristics of participants in each survey year. The mean ages (SD) of the participants were 59.4 (17.6) years in 2005, 61.0 (17.2) years in 2006, 63.6 (16.4) years in 2007, 64.5 (15.9) years in 2008, and 62.7 (16.9) years in 2009. The decrease in mean age in 2009 was due to the higher participation rate, especially among younger residents (Table 1). The 2005 characteristics of the 224 participants who participated in all 5 surveys are shown in Table 2. Their mean age in 2005 was 64.0 (13.7) years.

**Table 1.** Characteristics of participants, by survey year

	Number of participants				

	2005	2006	2007	2008	2009
	*n* = 1316	*n* = 667	*n* = 753	*n* = 541	*n* = 814
Age					
≤64 years	715 (54.3%)	329 (49.3%)	344 (45.7%)	228 (42.1%)	401 (49.3%)
≥65 years	601 (45.7%)	338 (50.7%)	409 (54.3%)	313 (57.9%)	413 (50.7%)

Sex					
Male	652 (49.5%)	326 (48.9%)	351 (46.6%)	252 (46.6%)	388 (47.7%)
Female	664 (50.5%)	341 (51.1%)	402 (53.4%)	289 (53.4%)	426 (52.3%)

Job status					
Unemployed	409 (31.9%)	226 (35.0%)	228 (31.6%)	151 (29.2%)	202 (25.7%)
Other	873 (68.1%)	419 (65.0%)	493 (68.4%)	367 (70.9%)	583 (74.3%)
Data missing	34	22	32	23	29

Contact with neighbors					
Often	484 (37.4%)	239 (36.7%)	254 (35.5%)	200 (38.3%)	277 (35.2%)
Sometimes	546 (42.1%)	304 (46.7%)	336 (46.9%)	233 (44.6%)	347 (44.0%)
Not often	185 (14.3%)	80 (12.3%)	98 (13.7%)	61 (11.7%)	113 (14.3%)
Rarely	81 (6.3%)	28 (4.3%)	28 (3.9%)	28 (5.4%)	51 (6.5%)
Data missing	20	16	37	19	26

GHQ-12 score					
≤3 (normal)	645 (49.0%)	384 (57.6%)	475 (63.1%)	378 (69.9%)	539 (66.2%)
≥4 (psychological distress)	671 (51.0%)	283 (42.4%)	278 (36.9%)	163 (30.1%)	275 (33.8%)

Abbreviation: GHQ-12, 12-item General Health Questionnaire.

**Table 2.** The 2005 characteristics of the 224 participants who participated in all 5 surveys, 2005–2009

	Number of participants
Age	
≤64 years	96 (42.9%)
≥65 years	128 (57.1%)

Sex	
Male	104 (46.4%)
Female	120 (53.6%)

Job status	
Unemployed	79 (35.9%)
Other	141 (64.1%)
Data missing	4

Contact with neighbors	
Often	107 (47.8%)
Sometimes	88 (39.3%)
Not often	22 (9.8%)
Rarely	7 (3.1%)

GHQ-12 score	
≤3 (normal)	116 (53.2%)
≥4 (psychological distress)	102 (46.8%)
Data missing	6

Abbreviation: GHQ-12, 12-item General Health Questionnaire.

**Figure 1.**
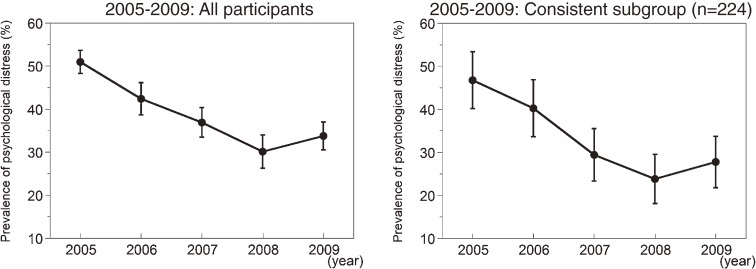
Secular changes in the prevalence of psychological distress after the Niigata-Chuetsu earthquake among all participants (left) and 224 participants who took part in all 5 surveys (right). Bars indicate 95% CIs.

The secular change in the prevalence of psychological distress in all participants, as well as in the 224 participants who participated in all 5 surveys, is illustrated in Figure 1. The overall prevalence of psychological distress (Figure 1, left) was highest in 2005, before decreasing to its lowest value in 2008. The decreasing trend from 2005–2009 was statistically significant (*P* < 0.0001). The secular trend in prevalence among the 224 participants (Figure 1, right) decreased in a similar manner (*P* < 0.0001).

**Figure 2.**
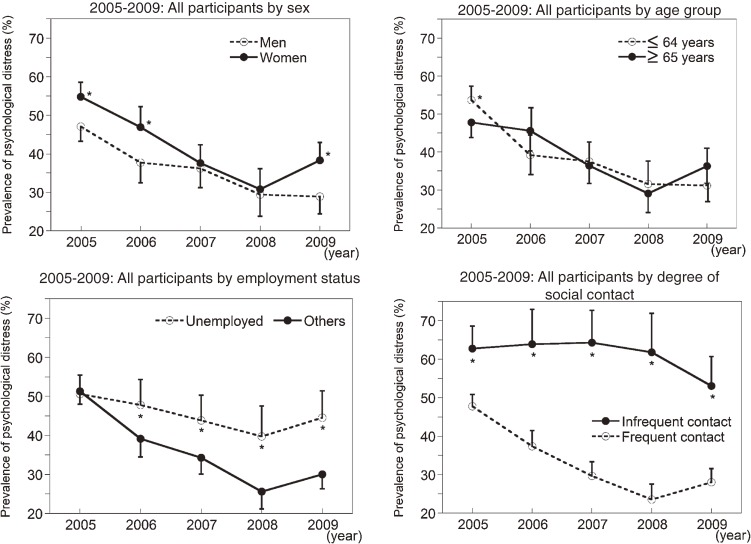
Secular changes in the prevalence of psychological distress after the Niigata-Chuetsu earthquake among participants stratified by sex (upper right), age group (upper left), job status (lower right), and level of social contact (lower left). Bars indicate 95% CIs. *P < 0.05 vs comparison group.

Secular changes in the prevalence of psychological distress in all participants according to sex, age (≤64 vs ≥65 years), jobless status (unemployed vs other), and degree of social contact (often/sometimes vs not often/rarely) are shown in Figure 2. A statistically significant decreasing trend in prevalence was noted between 2005 and 2009 for all groups except those reporting infrequent social contact (*P* = 0.0659). In relation to the respective comparison groups, the prevalence of psychological distress was higher among those reporting infrequent social contact (Figure 2, lower right) and unemployed participants (Figure 2, lower left) and tended to be higher among women than among men (Figure 2, upper left). There was no significant difference in prevalence between age groups (Figure 2, upper right).

Despite the overall decreasing trend in prevalence, psychological distress appeared to increase from 2008 to 2009 (Figure 1), although the change (+3.7%) was not statistically significant (*P* = 0.1590). However, changes in prevalence within some subgroups (Figure 2) were statistically significant, such as the increases in the prevalence of psychological distress among women (+7.5%; *P* = 0.0403) and participants aged 65 years or older (+7.2%; *P* = 0.0400). Multiple logistic regression analysis was conducted to identify independent factors related to change in distress status in 2008–2009 as an outcome variable (those without a diagnosis of psychological distress in 2008 and a new diagnosis of psychological distress in 2009 were coded as 1 [*n* = 38]; others were coded as 0 [*n* = 383]) in participants who participated in both the 2008 and 2009 examinations. This analysis revealed that none of the 4 possible predictors—ie, age (*P* = 0.1105), sex (*P* = 0.4550), employment status (*P* = 0.3360), and social contact (*P* = 0.7121)—were independently associated with change in prevalence in 2008–2009. Another multiple logistic regression analysis was conducted to identify independent factors related to psychological distress in 2009 (those who received a diagnosis of psychological distress were coded as 1 [*n* = 281]; others were coded as 0 [*n* = 558]) among participants who participated in the 2009 examinations. Psychological distress was independently associated with social contact (odds ratio [OR] = 2.93, 95% CI: 2.03–4.25, *P* < 0.0001), employment status (OR = 1.56, 95% CI: 1.07–2.27, *P* = 0.0219), and sex (OR = 1.70, 95% CI: 1.24–2.34, *P* = 0.0010), but not age (OR = 1.14, 95% CI: 0.81–1.60, *P* = 0.4517).

## DISCUSSION

This study presented findings related to recovery from psychological distress among adults living in a community hit by a large earthquake. We found that the prevalence of psychological distress decreased gradually and reached its lowest point at 4 years after the earthquake.

Although the short-term effects of earthquakes on mental health are well-documented, there are limited data on the long-term impacts of such events. Most prior studies assessed psychological disorders several months or a few years after earthquakes, but few studies investigated long-term outcomes (ie, >5 years after an earthquake).^12^^,^^13^ Bland et al^12^ assessed symptoms of psychological distress in people 7 years after the 1980 earthquake and found that levels of psychological distress were higher among those who had reported damage from the earthquake than among those who had not. Priebe et al^13^ assessed mental disorders in a randomly selected sample of 200 people who had experienced a 1997 earthquake in Italy 8 years before and concluded that the prevalence of mental disorders did not differ from that in comparable populations. Although the results of these 2 studies are inconsistent and not directly comparable with ours, the findings suggest that a large earthquake could affect the mental health of victims for a very long time, up to 7 years. Thus, to facilitate comparison with previous studies, further follow-up investigation in Yamakoshi is warranted.

The present subgroup analysis showed that the prevalence of psychological distress did not decrease over 5 years among those with poor social contact. This finding agrees with the results of a number of previous reports on earthquakes, which showed that disruption of social networks is a major factor in poor mental health. Oyama et al^11^ found that earthquake victims who were estranged from others in their community were at higher risk (OR = 7) for psychological distress 3 years after the Niigata-Chuetsu earthquake. Bland et al^14^ reported that those who were evacuated farther away from family or friends had higher levels of psychological distress 3 to 4 years after an earthquake in Italy. Kiliç et al^15^ showed that disruption of social networks due to relocation increased psychological distress 4 years after 2 severe earthquakes in Turkey.

The prevalence of psychological distress was higher among unemployed people, which was also noted in other studies.^11^^,^^16^ This may be due in part to financial burdens from house damage that adversely affect psychological health.^17^

The prevalence of psychological distress was higher among women than among men in 2005, 2006, and 2009. In general, common mental disorders are more prevalent among women,^18^ but a sex difference in the prevalence of GHQ-defined psychological distress was not found in a large Japanese population.^19^ A possible explanation for this higher prevalence in women may be the traditional, dependent roles of women in rural Japanese communities, including Yamakoshi.^11^^,^^20^ In such communities, women have a major role at home: they manage most of the housework and care for all family members, including their husband, children, and husband’s parents.^11^ Because of role change, post-earthquake burdens may have been greater for women than for men. The long-term higher prevalence of psychological distress in women versus men is noteworthy in relation to suicide rate after earthquakes. Long-term suicide rates after the Niigata-Chuetsu earthquake increased among women and decreased among men.^21^ The suicide rate among women may have been elevated because the prevalence of psychological distress in women was higher and increased over time.

The prevalence of psychological distress generally decreased after the earthquake, although there was an unexpected increase from 2008 to 2009. While multiple logistic regression analysis did not identify independent factors that contributed to this increase, there might have been stress-related factors beginning in 2009. Many people had been struggling to reestablish their lives and had difficulty continuing their daily tasks, which may have been reflected in greater psychological distress in 2009.

**Figure 3.**
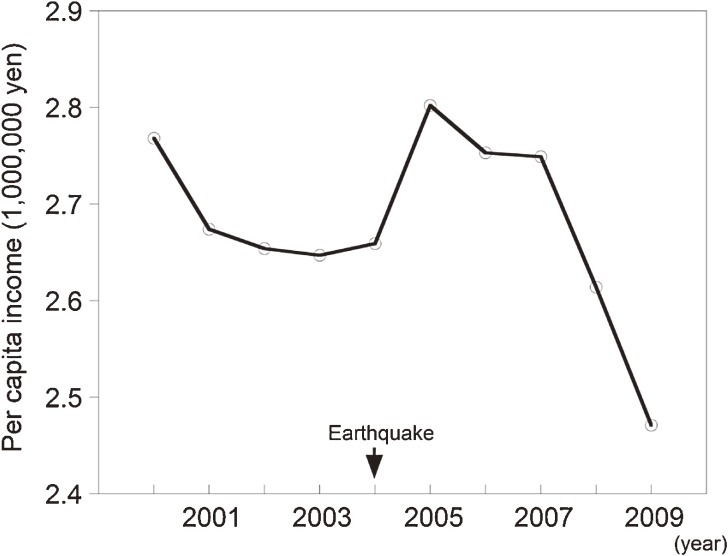
Secular change in per capita income in the disaster area (including Yamakoshi) of the Niigata-Chuetsu earthquake.

Many earthquake victims may have had difficulty rebuilding their lives due to financial losses, which are common after a large earthquake.^12^^,^^22^ In fact, the Niigata prefectural government reported declines in per capita income in the disaster areas after the Niigata-Chuetsu earthquake.^23^ Per capita income in the disaster areas decreased in 2008–2009, after rising in 2005–2007 as a result of temporary financial aid from the government (Figure 3).^23^ Financial problems caused by the earthquake may have been an important contributor to the worsening mental health of some Yamakoshi people 5 years later.

We evaluated the prevalence of psychological distress in an earthquake-stricken area rather than in a normal population. Few studies have used the GHQ-12 to investigate the prevalence of psychological distress in normal Japanese populations. Using the same GHQ-12 cutoff score, of 3/4, Shimbo et al^24^ reported that the prevalence rate of psychological distress was 18.9% to 20.0% in a randomly selected general population (*n* = 600) aged 20 to 69 years. This suggests that the prevalence of psychological distress in the present population was higher than that in a normal Japanese population.

A major limitation of this study is that participation rates differed throughout the 5-year study period; those between 2006 and 2008 (around 50%) were lower than those in 2005 and 2009. This discrepancy may have influenced prevalence rates. Secondly, we did not assess psychological distress before the earthquake and thus cannot determine whether the prevalence of psychological distress returned to pre-earthquake levels. Finally, the residents of Yamakoshi are mostly elderly adults in a rural area and, hence, generalization of our results requires caution. Our findings might not be applicable to younger populations in urban areas. Despite these limitations, data on long-term changes in post-earthquake psychological status among people living in a community near an earthquake epicenter are rare and worth reporting.

In summary, the overall prevalence of psychological distress steadily decreased during the 4-year period after the earthquake; however, prevalence tended to increase between the fourth and fifth years after the earthquake. In addition, the prevalence of psychological distress did not decrease among those who reported poor social contact. Rebuilding of the lives of earthquake victim is ongoing; thus, their mental health status should be closely monitored.

## ONLINE ONLY MATERIALS

Abstract in Japanese.
