# Supplementary material for: Psychological Recovery 5 Years After the 2004 Niigata-Chuetsu Earthquake in Yamakoshi, Japan
Source: J Epidemiol. 2014 Mar 5;24(2):125–31. doi: 10.2188/jea.JE20130097 (PMC3956692; doi:10.2188/jea.JE20130097)
Supplement: Abstract in Japanese. [file je-24-125-s001.pdf]

中越地震後５年における山古志住民の心理的苦痛の回復

中村和利 [1], 北村香織 [1], 染矢俊幸 [2,3]
[1] 新潟大学大学院医歯学総合研究科環境予防医学分野
[2] 新潟大学大学院医歯学総合研究科精神医学分野
[3] 新潟こころのケアセンター（新潟県精神保健福祉協会）

抄録
背景: 2004 年に発生した中越地震は多くの被害をもたらした。本研究の目的は地震後５年間の被災者における心理的苦痛の長期的変化を評価することである。
方法:対象者は１８歳以上の山古志（震源近くの自治体）の住民であった。地震後の５年間に自記式質問票による調査が毎年行われた。回答率に関して、2005 年は 1316/1841（71.5%）、2006 年は 667/1381（48.3%）、2007 は 753/1451（51.9%）、2008 年は 541/1243（43.5%）、2009 年は 814/1158（70.3%）であった。質問票により、性、年齢、職業、社会的ネットワーク、精神状態に関する情報を得た。心理的苦痛は General Health Questionnaire１２項目版 （GHQ-12）を用いて評価し、合計点４点以上を心理的苦痛ありと定義した。
結果: 全体として、心理的苦痛の有病率は 2005 年（51.0%）から 2008 年（30.1%）に徐々に低下した （P<0.0001）が、2008 年から 2009 年は上昇傾向 （P=0.1590）であった。サブグループ解析により、社会的コンタクトの悪いグループでは 5 年間の有病率は低下が見られなかった （P=0.0659）。また、女性（+7.5%, P=0.0403）および 65 歳以上の群（+7.2%, P=0.0400）では、2008 年から 2009 年の有病率の上昇が有意であった。
結論: 山古志住民の心理的苦痛は地震後４年間に徐々に低下したが、その後は上昇傾向であった。地震被災者の生活の復興は本研究期間を超えて続いており、彼らのメンタルヘルスは引き続き評価・維持されるべきである。

キーワード：疫学研究、地震、メンタルヘルス、心理的苦痛、社会的支援
